# The multifaceted functions of SPC25 in cancer: from molecular pathways to targeted therapy

**DOI:** 10.3389/fmed.2025.1550901

**Published:** 2025-05-07

**Authors:** Yigang Jin, Minjie Chen, Fei Chen, Zhaofeng Gao, Xiaoping Li, Lingyu Hu, Dandan Cai, Siqi Zhao, Zhengwei Song

**Affiliations:** ^1^Department of Urology, The Second Affiliated Hospital of Jiaxing University, Jiaxing, Zhejiang, China; ^2^Department of Surgery, The Second Affiliated Hospital of Jiaxing University, Jiaxing, Zhejiang, China

**Keywords:** SPC25, cancer progression, biomarker, therapeutic target, NDC80 complex

## Abstract

Spindle Pole Body Component 25 (SPC25), a critical component of the NDC80 kinetochore complex, plays an essential role in maintaining chromosomal stability during mitosis. Recent studies have revealed its aberrant expression in various cancers, highlighting its potential as both a diagnostic biomarker and a therapeutic target. This review provides a comprehensive analysis of the molecular mechanisms underlying SPC25’s involvement in tumorigenesis, including its regulation of cell cycle progression and interaction with key oncogenic pathways. Furthermore, we discuss its prognostic significance across different cancer types and its potential impact on therapy resistance. The emerging evidence underscores SPC25’s multifaceted role in cancer biology, offering novel insights into its clinical applications. We conclude by exploring future research directions, emphasizing the need for in-depth studies to unravel the precise molecular functions of SPC25 and its therapeutic potential in cancer treatment.

## 1 Introduction

Cancer remains one of the leading causes of morbidity and mortality worldwide, characterized by its heterogeneity and complexity at the molecular, cellular, and clinical levels (1–3). Spindle Pole Body Component 25 (SPC25), a core subunit of the NDC80 kinetochore complex, is essential for accurate chromosome alignment and segregation during mitosis (4–6). By linking microtubules to kinetochores, SPC25 ensures proper attachment and tension generation, critical for the maintenance of genomic stability (7, 8). Aberrant expression of SPC25 disrupts these processes, leading to aneuploidy, which are frequently observed in malignant cells (9–11). Emerging evidence suggests that SPC25 is not merely a passive player in cell division but also participates in oncogenic signaling pathways, making it an intriguing target for cancer research (4, 9, 12).

In recent years, studies have highlighted the overexpression of SPC25 in various cancers, including lung, breast, gastric, and colorectal cancers (CRC) (10, 13–15). Its elevated levels are often associated with aggressive tumor phenotypes, poor prognosis, and resistance to therapy, underscoring its potential as a diagnostic and prognostic biomarker (15–17). Moreover, the role of SPC25 in modulating the tumor microenvironment (TME) and its crosstalk with other cellular pathways have further expanded its relevance in cancer biology (18, 19). Despite these advances, a comprehensive understanding of SPC25’s molecular mechanisms and clinical implications in cancer remains incomplete.

**Figure 1:**
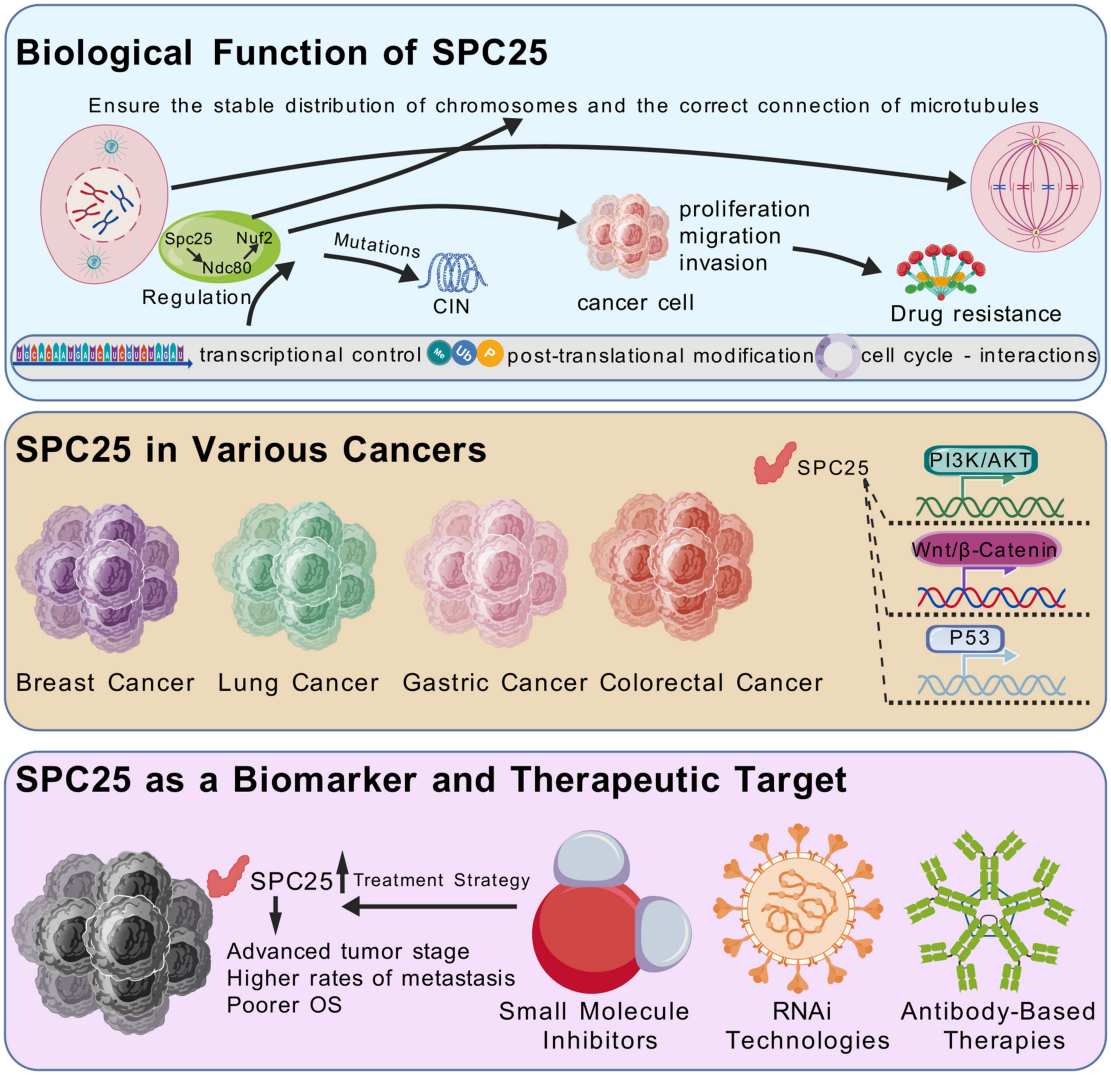
SPC25 is an important part of the NDC80 centromere complex. SPC25 ensures the stability of the centromate-microtubule attachment through interactions with other NDC80 complex components, thus maintaining chromosome integrity. Its function is strictly regulated by multiple layers of transcriptional regulation, post-translational modification, and cell cycle dependent interactions. SPC25 not only plays an important role in chromosome stability and cell division, but also interacts with a variety of key signaling pathways for tumor cell proliferation, survival and metastasis. These pathways include the Wnt/*β*-catenin pathway, PI3K/AKT pathway, and p53 signaling pathway. Thus, dysregulation of SPC25 in various cancers makes it a promising candidate for biomarker development and targeted therapy.

This review aims to provide a detailed examination of the current knowledge regarding SPC25 in cancer biology. We begin by exploring the structural and functional characteristics of SPC25 within the NDC80 complex, followed by its dysregulation in different tumor types and its impact on tumor progression. Furthermore, we discuss its emerging roles in cancer progression, including its involvement in oncogenic pathways and therapy resistance. Also, we highlight the potential of SPC25 as a biomarker and therapeutic target (Figure 1). Finally, we discuss future research directions and how to take full advantage of its clinical potential.

By consolidating existing evidence, this article seeks to bridge the gap between basic research and clinical applications, offering novel insights into the role of SPC25 in cancer and its implications for the development of innovative diagnostic and therapeutic strategies.

## 2 Biological function of SPC25

SPC25 is an essential component of the NDC80 kinetochore complex, which plays a central role in mitosis by mediating the attachment of chromosomes to the spindle microtubules (5, 12, 20–22). The NDC80 complex, consisting of NDC80, SPC25, SPC24, and HEC1, facilitates the proper alignment and segregation of chromosomes during cell division (23–27). SPC25, through its interactions with other NDC80 complex components, ensures the stability of kinetochore-microtubule attachments, thus maintaining chromosomal integrity (5).

### 2.1 SPC25 in mitosis and chromosome segregation

Accurate chromosome segregation during mitosis is critical for cell division and genetic stability (28, 29). SPC25 plays an essential role in this process by facilitating the proper attachment of chromosomes to the mitotic spindle microtubules through its interaction with the NDC80 complex. The NDC80 complex, which is a key component of the kinetochore, bridges the inner kinetochore to the outer microtubule-binding layer, ensuring stable chromosome alignment at the metaphase plate and segregation during anaphase (30). The NDC80 complex comprises four subunits, including SPC25, SPC24, NDC80, and NUF2, and is structured as an elongated rod with globular domains at each end, critical for its function in mitosis (31).

SPC25 interacts with SPC24 to stabilize the NDC80 complex, allowing it to maintain stable association with microtubules throughout mitosis (4, 25). The C-terminal domain of SPC25 directly binds to microtubules, facilitating dynamic regulation during cell division. This microtubule-binding interface involves a cooperative, electrostatic interaction, which is essential for kinetochore-microtubule attachment (32). Disruption of SPC25 function or its binding to microtubules leads to defective chromosome alignment and segregation (33–35).

Recent structural insights into the NDC80 complex have revealed that SPC25, along with SPC24, forms a globular structure that directly interacts with the microtubules, a critical process for accurate chromosome segregation. Mutations or malfunctions in SPC25 hinder this interaction, resulting in misaligned chromosomes and aneuploidy (20, 31).

### 2.2 Regulation of SPC25

The function of SPC25, as a core component of the NDC80 complex, is tightly regulated through multiple layers, including transcriptional control, post-translational modifications, and cell cycle-dependent interactions. Understanding these regulatory mechanisms is crucial to uncovering the full range of SPC25’s roles in mitosis.

The expression of SPC25 is primarily regulated at the transcriptional level, where various transcription factors likely play a role. Studies have suggested that certain oncogenic signaling pathways, such as PI3K/AKT and Wnt/*β*-catenin, can modulate the transcription of genes involved in mitotic progression, including SPC25 (17, 36–38). These pathways, which are frequently dysregulated in cancer, could lead to the overexpression of SPC25, contributing to tumor progression. Further studies are needed to identify the specific transcription factors or regulatory elements that control SPC25 expression in different cancer types, as well as how these pathways integrate with the cellular environment.

SPC25 is subject to various post-translational modifications, which are critical for its function in mitosis. Phosphorylation is one of the most prominent modifications regulating SPC25’s activity. Phosphorylation of SPC25 during mitosis is likely mediated by kinases such as Cyclin-dependent kinases and Aurora B kinase, which are essential for proper chromosome alignment and segregation (39, 40). Phosphorylation could regulate SPC25’s interaction with other components of the NDC80 complex or with microtubules, thus modulating its function during the mitotic phase (20, 36, 39–41).

Ubiquitination is another important modification that may control SPC25 stability and its turnover. The precise regulation of SPC25 degradation is crucial for maintaining mitotic fidelity, as premature degradation or accumulation of SPC25 could lead to chromosome missegregation (42, 43). But there is still no research to explore this in depth. Additionally, acetylation, methylation, and SUMOylation may also play roles in regulating SPC25 activity, although these modifications require further exploration. Such modifications can affect SPC25’s interactions with other mitotic regulators and may provide another layer of control in the maintenance of chromosomal stability.

SPC25’s activity is intricately linked to the progression of the cell cycle, with its function being highly dependent on cell cycle-dependent interactions with key mitotic proteins (4, 44). During mitosis, SPC25 forms stable interactions with its binding partners in the NDC80 complex, including SPC24, NDC80, and NUF2, to ensure proper chromosome alignment and segregation. These interactions are dynamically regulated, with SPC25 being recruited to the kinetochore at the onset of mitosis and dissociating during telophase (4, 5, 45, 46).

At specific stages of the cell cycle, SPC25 may also interact with other regulatory proteins to modulate its function. For example, interactions with Aurora B kinase during mitosis help to regulate the attachment of kinetochores to microtubules, ensuring proper chromosome alignment and preventing premature segregation (8, 39, 40). Similarly, SPC25 may interact with checkpoint proteins, such as Mad2, to monitor and ensure that all chromosomes are properly attached to the spindle before anaphase proceeds (33, 47). These interactions are essential for maintaining genomic stability, and any disruption of the cell cycle-dependent regulation of SPC25 can contribute to cancer progression.

In summary, the regulation of SPC25 through transcriptional control, post-translational modifications, and cell cycle-dependent interactions is essential for its role in mitosis and chromosomal stability. Dysregulation of these mechanisms can lead to SPC25 overexpression or malfunction, which contributes to cancer progression. Understanding these regulatory processes in greater detail will offer valuable insights into the therapeutic potential of targeting SPC25 in cancer.

### 2.3 SPC25 and cancer progression

Beyond its role in mitosis, SPC25 also contributes to the progression of cancer by influencing various cellular processes such as proliferation, migration, and invasion (48, 49). The overexpression of SPC25 in cancer cells enhances cell cycle progression, particularly through its involvement in the G2/M transition (50). SPC25 stabilizes the NDC80 complex and promotes the efficient separation of chromatids during mitosis, which accelerates cell division. This, in turn, facilitates the rapid proliferation of cancer cells.

In addition to promoting cell proliferation, SPC25 has been implicated in enhancing cancer cell migration and invasion, two key processes involved in tumor metastasis (15, 51, 52). Research has demonstrated that SPC25 interacts with signaling pathways known to regulate cell migration, such as the PI3K/AKT and MAPK pathways (36, 37, 53). These interactions may contribute to the ability of cancer cells to migrate through tissues and invade distant organs. Furthermore, SPC25’s role in maintaining genomic stability and promoting cell proliferation may help establish a TME conducive to the survival and expansion of cancer cells (54, 55).

### 2.4 SPC25 and chemotherapy resistance

In addition to its involvement in tumor progression, SPC25 plays a critical role in cancer cell resistance to chemotherapy and targeted therapies (14, 17, 56, 57). Chemotherapy agents that target microtubules, such as paclitaxel, work by stabilizing microtubules and disrupting their dynamic instability, leading to mitotic arrest and cell death (58, 59). However, cancer cells often develop resistance to these therapies through various mechanisms, including the dysregulation of mitotic regulators like SPC25.

SPC25 overexpression has been shown to enhance the stability of kinetochore-microtubule attachment, which can result in a more stable microtubule-kinetochore interaction (5, 9). While this stabilizing effect may be synergistic with the actions of paclitaxel in promoting microtubule stabilization, it can paradoxically reduce the effectiveness of paclitaxel by potentially mitigating the disruption of microtubule dynamics that is critical for drug efficacy. The enhanced stability of kinetochore-microtubule attachments may make it more difficult for paclitaxel to further disrupt the mitotic spindle, thereby impairing the drug’s ability to induce mitotic failure.

Additionally, SPC25 may contribute to resistance by regulating DNA repair mechanisms. Cancer cells with elevated SPC25 levels may be better equipped to repair DNA damage induced by chemotherapy, allowing them to survive and proliferate despite treatment (14, 60). This ability to overcome chemotherapy-induced cell death highlights SPC25 as a potential target for overcoming therapy resistance in cancer treatment.

## 3 SPC25 in different cancers: recent advances

Numerous studies have investigated the expression and functional implications of SPC25 across various tumor types, including breast cancer, lung cancer, gastric cancer (GC), and CRC. These studies have provided valuable insights into the clinical significance and molecular mechanisms of SPC25, as well as its interactions with key signaling pathways. A summary of these relevant studies is presented in Table 1, which highlights the expression levels, prognostic value, and functional roles of SPC25 in these cancers. This section will review the current knowledge regarding these aspects of SPC25, with a focus on its contribution to cancer progression and its potential as a therapeutic target.

**Table 1:** Research progress of SPC25 in tumor.

Cancer type	Function and mechanism	Clinical significance	Ref
Breast cancer	SPC25 is upregulated in BC tissues and is associated with increased recurrence rates and decreased survival in patients. High SPC25 expression correlates with shorter distant metastasis-free survival, relapse-free survival, and OS. Single-cell analysis and functional assays indicate that SPC25 promotes BC cell proliferation by regulating the cell cycle and DNA damage repair, while its knockout inhibits proliferation. miRNAs, circRNAs, RNA-binding proteins, transcription factors, and immune factors may interact with SPC25 mRNA to further drive BC development and progression.	DNA methyltransferase inhibitors and transcription factor inhibitors may improve survival in BC patients by targeting SPC25.	(60)
Breast cancer	Luminal progenitor and basal stem cells are susceptible to genetic and epigenetic modifications that promote oncogenic transformation and tumorigenic potential. In a mouse model, combined treatment with the DNMT inhibitor 5-azacytidine and the HDAC inhibitor butyrate significantly reduced CSC abundance and improved survival rates. RNA-seq analysis revealed that this treatment inhibited growth-promoting molecules such as RAD51AP1 and SPC25, which play crucial roles in DNA damage repair and centromere assembly. RAD51AP1 and SPC25 are overexpressed in human breast tumors and are associated with reduced OS.	Breast CSCs are intrinsically sensitive to genetic and epigenetic modifications, and epigenetic-based therapies may significantly impact their behavior. Further research is needed on DNMT and HDAC co-inhibition in refractory or drug-resistant breast cancer.	(14)
Breast cancer	The study identified six mRNAs to construct a prognostic risk scoring system, including four upregulated mRNAs (RDH16, SPC25, SPC24, and SCUBE3) and two downregulated mRNAs (DGAT2 and CCDC69).	The risk scoring system helps screen high-risk HER2-positive breast cancer patients, promoting early diagnosis and personalized treatment, indicating that these mRNAs play a key role in the progression of HER2-positive breast cancer.	(56)
Clear cell renal cell carcinomas	The mRNA expression levels of AURKA, AURKB, BIRC5, BUB1, CDC20, NEK2, and SPC25 are significantly higher in CIMP-positive RCC compared to CIMP-negative RCC.	The data suggest that abnormalities in the spindle checkpoint pathway contribute to the development of CMP-positive renal cancer.	(16)
Endometrial cancer	SPC25 is differentially expressed in UCEC and identified as a risk factor for poor OS, DSS, and PFI, particularly in various clinical subtypes. SPC25 and its co-expressed genes are mainly involved in cell cycle and cell division pathways. Low CpG methylation in SPC25 leads to its high expression, correlating with poor prognosis. Additionally, SPC25 may influence UCEC progression and immunotherapy outcomes by modulating immune molecules and chemokines, altering immune cell infiltration in the TME.	SPC25 is a predictive biomarker and potential therapeutic target for UCEC.	(54)
Endometrial carcinoma	Six genes (DEPDC1, FAM83D, NCAPH, SPC25, TPX2, and TTK) are upregulated in endometrial cancer and associated with higher stage, age, and grade. Their pathogenicity may involve copy number alterations and reduced methylation levels. GSEA indicates that these genes are closely linked to the cell cycle.	These findings provide new insights for disease treatment. 4o mini	(106)
Epithelial ovarian cancer	SPC25 acts as a scaffold to coordinate the assembly of the SPC25/RIOK1/MYH9 complex, triggering RIOK1-mediated MYH9 Ser1943 phosphorylation. This promotes MYH9 nuclear accumulation and activates Wnt/β-catenin signaling. The inhibitory peptide CBP1 disrupts this complex, reducing Wnt/β-catenin activity, weakening the CSC phenotype, and enhancing the efficacy of platinum therapy in vitro, in vivo, and in patient-derived organoids.	Targeting the SPC25/RIOK1/MYH9 axis may enhance platinum sensitivity and improve survival in EOC patients.	(17)
Head and neck cancer	New mitotic mechanisms are significantly associated with cisplatin resistance, with MSRB3, RHEB, ULBP1, and SPC25 correlating with poor prognosis in HNC patients. The study further shows that silencing SPC25 significantly suppresses tumor stemness and reduces cisplatin resistance. The natural extract Celastrol effectively inhibits SPC25 expression and reverses the cisplatin resistance phenotype.	Developing SPC25 inhibitors, such as celastrol, may offer a new strategy for platinum sensitization in refractory HNC.	(94)
Hepatocellular carcinoma	SPC25 promotes DNA damage in liver cancer cells, activating the DNA-PK/Akt/Notch1 signaling cascade. The NICD/RBP-Jκ complex directly targets SOX2 and NANOG transcriptionally, regulating HCC cell proliferation and self-renewal.	This provides biomarkers for predicting HCC progression and poor survival, as well as potential therapeutic targets for HCC patients.	(9)
Hepatocellular carcinoma	SPC25 is highly expressed in HCC and is associated with poor prognosis and metastasis. Silencing SPC25 significantly inhibits HCC cell invasion and migration. Gene expression profiling reveals that SPC25 primarily affects genes related to ECM-integrin interactions, including the ITGB4. Upregulation of ITGB4 partially reverses the decrease in invasion and migration caused by SPC25 silencing. Deletion of SPC25 and ITGB4 reduces phosphorylation of FAK, PI3K, and AKT, downstream elements of the integrin pathway.	SPC25 plays an important role as a prognostic marker and metastasis promoter in HCC, with its mechanism partially elucidated, suggesting it as a biomarker and therapeutic target for HCC treatment.	(36)
Hepatocellular carcinoma	SPC25 mRNA expression is upregulated in HCC tissues, and its transcriptional level is significantly higher in Asian patients compared to Caucasians. SPC25 promotes liver cancer cell proliferation in vitro and tumor growth in vivo by accelerating the cell cycle. Several transcription factors, miRNAs, and immune cells potentially interacting with SPC25 have been identified.	Increased SPC25 expression is associated with poor prognosis in HCC and enhances cell proliferation, making it a valuable prognostic marker and a new therapeutic target for liver cancer.	(107)
Hepatocellular carcinoma	Knockdown of SPC25 significantly inhibits HCC cell proliferation and metastasis, while increasing the protein levels of components in the p53 pathway.	SPC25 is a potential tumor promoter in HCC, possibly acting through the p53 pathway.	(70)
Hepatocellular carcinoma	SPC25 is significantly upregulated in HCC (*p* < 0.001) with high diagnostic value (AUCs of 0.969 and 0.945). High SPC25 expression correlates with poor prognosis (*p* < 0.001) and independently predicts OS, with a nomogram consistency index higher than the AJCC staging system.	SPC25 is upregulated in HCC and independently predicts poor OS, making it an effective diagnostic and prognostic biomarker. The SPC25-based nomogram predicts HCC prognosis more accurately and usefully than the AJCC staging system.	(108)
Hepatocellular carcinoma	PRC1 regulates the expression and function of FANCI, SPC25, KIF11, and KIF23 through the Wnt signaling pathway.	PRC1 is a new Wnt target that enhances Wnt signaling through a positive feedback loop, promoting early HCC recurrence.	(38)
Hepatocellular carcinoma	Pseudotime analysis identified CDCA8, CENPA, SPC25, and TTK as central genes in HCC progression, associated with cellular senescence.	The study developed a prognostic model for HCC and identified new potential targeted therapies.	(90)
Hepatocellular carcinoma	A risk score based on the expression of CCNB2, DYNC1LI1, KIF11, SPC25, and KIF18A was constructed through stepwise regression and validated in TCGA and ICGC cohorts for predicting OS in HCC patients.	This feature predicts the OS of HCC patients well.	(109)
Hepatocellular carcinoma	Six prognosis-related AFs (GRM8, SPC25, FSD1L, SLC386A, FAM72A, and SLC39A10) were identified through LASSO Cox regression, providing the basis for a new prognostic risk model.	This model provides a reference for clinical management of HCC patients in the era of precision medicine.	(110)
Hepatocellular carcinoma	Five significantly upregulated biomarkers (SPC25, NUF2, MCM2, BLM, and AURKA) were found to predict survival risk in HCC patients, and a risk score model was established. The patients with higher risk scores had the worse survival compared with lower ones.	The study provides new prognostic biomarkers and models for HCC to enhance the understanding of the disease.	(111)
Lung adenocarcinoma	SPC25 is highly expressed in LUAD, correlating with staging and prognosis. GSEA analysis revealed that high SPC25 expression is involved in cell cycle and glycolysis pathways. Suppressing SPC25 significantly affects LUAD cell proliferation, migration, and apoptosis. A ceRNA network comprising SPC25 and SNHG15/hsa-miR-451a/SPC25 was successfully constructed, predicting its function.	SPC25 is a biomarker and therapeutic target for LUAD treatment.	(52)
Lung adenocarcinoma	PLEK2 is upregulated in LUAD and associated with poor prognosis. Knockdown of PLEK2 significantly inhibits LUAD cell proliferation and migration while enhancing apoptosis, and tumor growth in mice injected with PLEK2-silenced cells is impaired. Gene expression profiles and Co-IP analysis show a direct interaction between PLEK2 and SPC25, and downregulation of SPC25 also impairs cell proliferation and migration. Additionally, PI3K/AKT signaling activation is essential for the malignant phenotype induced by PLEK2 in LUAD.	The study highlights the carcinogenic potential of PLEK2 and SPC25 in LUAD, demonstrating their utility as prognostic markers and therapeutic targets.	(37)
Lung adenocarcinoma	The expression of NDC80, NUF2, SPC24, and SPC25 is significantly elevated in LUAD tumors, with diagnostic value (AUC > 0.900). High expression of NDC80 and SPC25 is associated with poor OS, and their combined expression predicts worse prognosis.	The NDC80 complex genes may serve as early diagnostic and prognostic markers for LUAD. The combined detection of NDC80, NUF2, SPC24, and SPC25 could become a new direction for diagnosis and targeted gene therapy.	(64)
Lung adenocarcinoma	Through protein-protein interaction network analysis, 13 hub genes (ANLN, RACGAP1, KIF4A, KIF20A, KIF14, ASPM, CDK1, SPC25, NCAPG, MKI67, HJURP, EXO1, HMMR) were identified, and their high expression is significantly associated with poor prognosis in LUAD patients.	The CNN-Cox model can effectively extract prognostic factors and biologically significant gene features.	(112)
Lung cancer	In IPF-LC, the mutation frequencies of CADM1 and SPC25 were 47% and 53%, respectively. About one-third of the cases had both mutations. Pathway analysis indicated that these genes are involved in the TGF-β1 signaling pathway. Mutations decreased CADM1 expression and increased SPC25 expression, promoting epithelial-to-mesenchymal transition and cell proliferation in lung cancer cells. Paclitaxel and DNMT1 inhibitors inhibited SPC25 expression.	Mutations in CADM1 and SPC25 may serve as new diagnostic markers and therapeutic targets for IPF-LC.	(65)
Lung cancer	SPC25 is upregulated in both stiff matrix and tumor tissues. Under stiff matrix conditions, shRNA knockdown of SPC25 significantly inhibits H1299 cell proliferation and downregulates Cyclin B1 expression, whereas under soft matrix conditions, SPC25 silencing does not affect cell proliferation rate.	This finding reveals a new key molecule linking EMC stiffening to cancer progression.	(113)
Non-small cell lung cancer	SPC25 is highly expressed in LUAD and LUSC tissues. Overexpression of SPC25 significantly enhances the CSC properties and invasiveness of A549 cells, while silencing SPC25 affects these properties in A549 cells. High SPC25 expression is an independent poor prognostic factor for OS and relapse-free survival (RFS) in LUAD patients, but no independent prognostic value of SPC25 is observed in LUSC patients.	Upregulation of SPC25 enhances CSC characteristics in LUAD and independently predicts lower survival rates for this histological subtype.	(15)
Oral squamous cell carcinoma	Hub genes VRK1, NUP37, HMMR, SPC25, and RUVBL1 are associated with oral cancer and show differential expression between tumor and normal tissues. Patients with high expression of these genes have poorer prognosis.	They may contribute to improving patient survival and prognosis, which requires further analysis.	(71)
Pan-cancer	SPC25 transcription and protein expression are elevated in most cancers and predict survival in patients with ACC, CESC, KIRC, KIRP, LIHC, LUAD, MESO, STAD, THYM, and UCEC. SPC25 is also closely associated with TMB, MSI, immune-related pathways, immune cell infiltration, immune checkpoint genes, and other immune regulation-related genes.	The study explores the potential of SPC25 as a pan-cancer prognostic and immunotherapy marker, providing new evidence for cancer treatment.	(19)
Prostate cancer	SPC25 expression is higher in PrC samples than in paired normal prostate tissues, and PrC patients with high SPC25 have a lower OS rate compared to those with low SPC25. SPC25+ cells form more tumor spheres, exhibit stronger resistance to docetaxel-induced apoptosis, and generate larger tumors with higher frequency in adoptive transfer.	SPC25 may be highly expressed in CSC-like cells of PrC and is a potential therapeutic target.	(114)
Prostate cancer	SPC25 is significantly upregulated in PRAD tissues and cells, correlating with poor prognosis. It enhances cancer cell viability, promotes glycolysis, and inhibits ferroptosis. Overexpression of SPC25 increases extracellular acidification, glucose uptake, and lactate secretion, while suppressing oxygen consumption. 2-DG counteracts some of SPC25's effects, alleviating changes in ferroptosis markers.	This study provides reference for predicting the prognosis of PRAD and finding new therapeutic targets.	(115)
Prostate cancer	In DHT-treated androgen-insensitive PCa cells, SPC25 is significantly decreased. DHT treatment increases growth, invasion, and metastasis in androgen-sensitive PCa cells, but reduces these functions in androgen-insensitive cells, mainly because DHT regulates SPC25 expression at the transcriptional level.	Androgen treatment inhibits CRPC growth, invasion, and metastasis by regulating SPC25, making SPC25 a promising target for CRPC therapy.	(57)
Prostate cancer	SPC25 may activate Egr1 to induce PDGF expression in PrC, and the secreted PDGF signals to TAMs through PDGFR on macrophages and polarized macrophages, potentially promoting PrC cell growth via TGF-β1 production and secretion.	SPC25 promotes PrC growth by triggering crosstalk between TAM and PrC cells via the SPC25/PDGF/PDGFR/TGFβ1 signaling pathway.	(18)
Prostate cancer	This study found significant upregulation of SPC25 in PCa. SPC25 knockdown inhibited proliferation, increased G2/M-phase cells, and promoted apoptosis. Bioinformatics analysis showed SPC25 regulates proliferation, apoptosis, invasion, and TGF-β pathways. TCGA data revealed SPC25 upregulation in advanced PCa, and lower SPC25 expression was linked to better survival.	SPC25 has oncogenic effects in prostate cancer and may serve as a new diagnostic and therapeutic target.	(50)

### 3.1 SPC25 in various cancers

#### 3.1.1 Breast cancer

Breast cancer, one of the most common malignancies worldwide, is characterized by high genomic instability, which plays a significant role in tumor progression (61–63). Studies have shown that SPC25 is overexpressed in a significant proportion of breast cancer cases. Overexpression of SPC25 has been associated with advanced tumor stages, higher histologic grades, and worse overall survival (OS) and disease-free survival in breast cancer patients (54, 56, 60). For instance, a study by Wang et al. demonstrated that SPC25 expression was significantly higher in breast cancer tissues compared to normal adjacent tissues, and its high expression was correlated with poor prognosis. Additionally, SPC25 overexpression may be linked to tumor evolution and resistance to treatment (11, 14, 56).

#### 3.1.2 Lung cancer

Lung cancer is another malignancy in which SPC25 plays a pivotal role. Both non-small cell lung cancer (NSCLC) and small cell lung cancer exhibit elevated SPC25 levels. In NSCLC, SPC25 overexpression correlates with tumor size, lymph node metastasis, and poor prognosis (13, 15, 37). SPC25 is thought to promote lung cancer progression by regulating cell cycle progression and inhibiting apoptosis (52, 64, 65). For example, a study by Chen et al. found that SPC25 upregulation can increase cancer stem cell (CSC) properties in lung adenocarcinoma (LUAD) and independently predict poor survival in this histological subtype (15). Liu et al. showed that inhibiting SPC25 expression significantly affected the proliferation, migration and apoptosis of LUAD cells. Abnormal SPC25 expression levels can affect cell cycle progression, glycolysis capacity and regulation of ferroptosis (52). These findings suggest that SPC25 may serve as a potential biomarker for prognosis and a therapeutic target for lung cancer.

#### 3.1.3 Gastric cancer

GC is one of the leading causes of cancer-related mortality globally, and its prognosis remains poor due to late-stage diagnosis and metastasis (66–68). Recent studies have revealed that SPC25 is frequently overexpressed in GC tissues. SPC25 overexpression has been associated with more aggressive tumor phenotypes, including larger tumor size, increased lymphatic invasion, and higher rates of metastasis (10, 13). A study by Zeng et al. reported that after the knockout of SPC25, the viability and proliferation of GC cells were significantly reduced (10). This suggests that SPC25 contributes to GC progression by promoting tumor cell proliferation and migration through its involvement in cell cycle regulation and mitotic progression.

#### 3.1.4 Hepatocellular carcinoma

In hepatocellular carcinoma (HCC), SPC25 exhibits multifaceted oncogenic roles. Clinically, its high expression correlates with poor prognosis, metastasis, and early recurrence (36, 38). Mechanistically, SPC25 promotes DNA damage in HCC cells, activating the DNA-PK/Akt/Notch1 cascade. This triggers the NICD/RBP-Jκ complex to transcriptionally upregulate stemness factors SOX2 and NANOG, driving tumor proliferation and self-renewal (9). Additionally, SPC25 enhances metastasis by modulating ECM-integrin interactions: its silencing suppresses HCC cell invasion/migration via downregulating ITGB4, while rescuing ITGB4 restores FAK/PI3K/AKT phosphorylation, reactivating integrin signaling (36). Furthermore, SPC25 is integrated into a Wnt/PRC1-driven positive feedback loop – PRC1 (a Wnt target) transcriptionally regulates SPC25, KIF11, and KIF23, which collectively amplify Wnt signaling to accelerate HCC recurrence (38). These findings position SPC25 as both a prognostic biomarker and a multi-pathway therapeutic target in HCC. More studies are summarized in Table 1.

#### 3.1.5 Colorectal cancer

In CRC, SPC25 has been implicated in both the development and progression of the disease (13, 69). Elevated SPC25 expression has been linked to poor prognosis and aggressive features in CRC, including advanced tumor stage and distant metastasis. A study by Naoyuki et al. showed that SPC25 was significantly overexpressed in CRC tissue samples compared to adjacent normal tissue. Additionally, the expression levels of tumor/normal ratios of CDCA1, KNTC2, SPC24 and SPC25 correlated with each other in CRCs, and the cell growths after the small interfering RNA (siRNA)-mediated knockdown were significantly suppressed (13). SPC25 contributes to CRC progression by promoting cell cycle progression, inhibiting apoptosis, and enhancing cell migration. Furthermore, SPC25’s interaction with microtubules in the mitotic spindle facilitates the maintenance of genomic stability, which may be a hallmark of CRC.

### 3.2 SPC25 and its interaction with key signaling pathways

SPC25 not only contributes to chromosomal stability and cell division but also interacts with various critical signaling pathways involved in cancer cell proliferation, survival, and metastasis. These pathways include the Wnt/*β*-catenin pathway, PI3K/AKT pathway, and p53 signaling pathway, all of which are frequently dysregulated in cancer (17, 36–38, 70–72).

#### 3.2.1 Wnt/*β*-catenin signaling pathway

**Figure 2:**
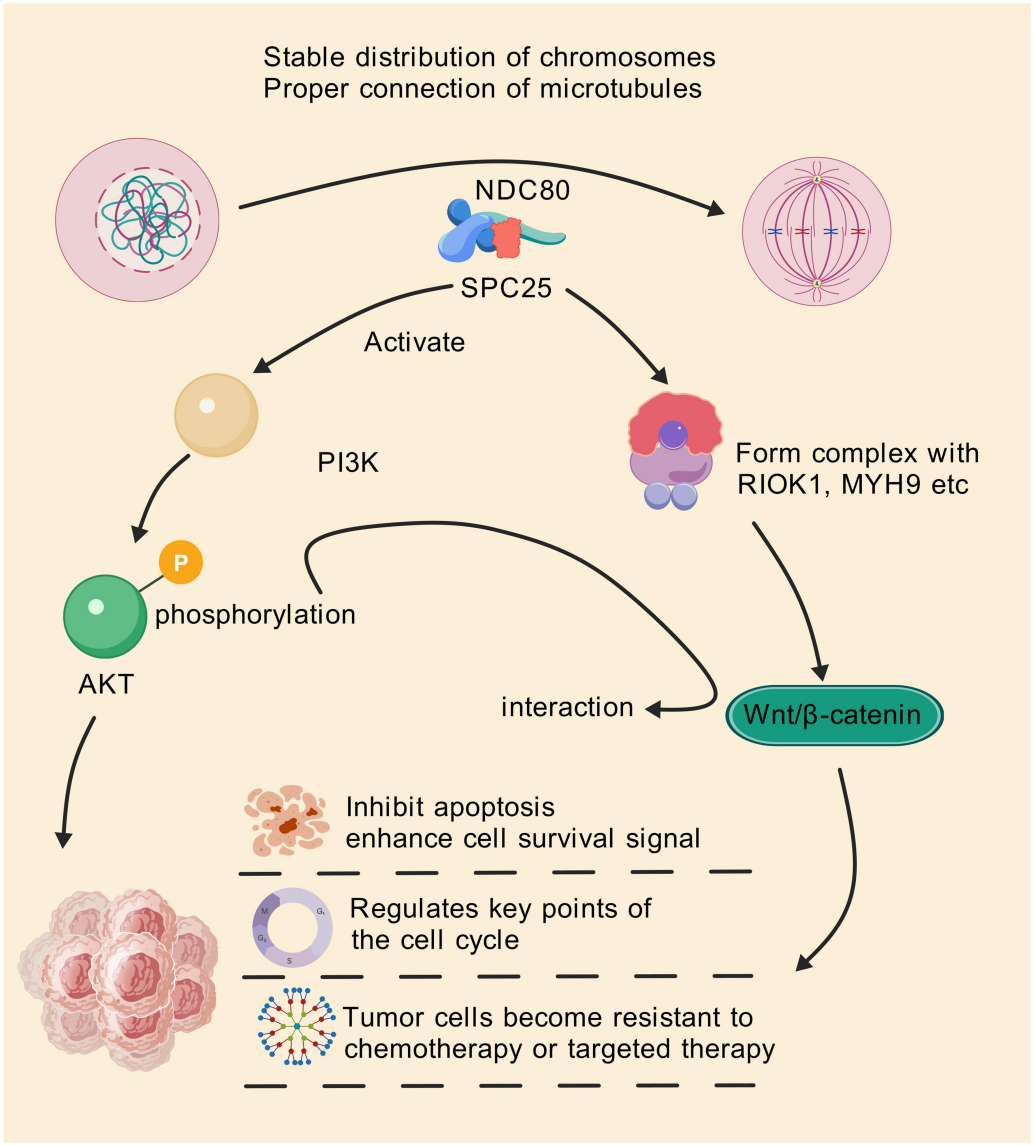
SPC25 can be used as a scaffolding platform to coordinate the assembly of the SPC25/RIOK1/MYH9 trimer complex, triggering RIOK1-mediated phosphorylation of MYH9 Ser1943. This prompts MYH9 to break away from the cytoskeleton and increase its nuclear accumulation, thereby enhancing CTNNB1 transcription and subsequent activation of the Wnt/β-catenin signaling pathway. In addition, SPC25 is also involved in abnormal activation of the PI3K/AKT pathway, which helps to improve cell survival and resist apoptosis. Through these pathways, SPC25 inhibits apoptosis of cancer cells and enhances survival signals of cancer cells, regulates key nodes in the cell cycle, and makes tumor cells resistant to therapy.

The Wnt/β-catenin signaling pathway is crucial for regulating cell proliferation, differentiation, and migration (73–75). Aberrant activation of Wnt signaling is commonly observed in many cancers, including colorectal, gastric, and breast cancers (76–78). SPC25 has been shown to interact with this pathway, promoting tumorigenesis by enhancing Wnt/β-catenin activity (Figure 2). In epithelial ovarian cancer, SPC25 acts as a scaffolding platform, orchestrating the assembly of an SPC25/RIOK1/MYH9 trimeric complex, triggering RIOK1-mediated phosphorylation of MYH9 at Ser1943. This prompts MYH9 to disengage from the cytoskeleton, augmenting its nuclear accumulation, thus potentiating CTNNB1 transcription and subsequent activation of Wnt/β-catenin signaling (17). It has also been reported that PRC1 controls the expression and function of wrrag such as FANCI, SPC25, KIF11, and KIF23 via Wnt signaling in hepatocellular carcinoma (HCC) (38).

#### 3.2.2 PI3K/AKT pathway

The PI3K/AKT signaling pathway is essential for regulating cell survival, growth, and metabolism (79–81). Aberrant activation of the PI3K/AKT pathway is a common feature of various cancers, including breast, lung, and colorectal cancers (80, 82).SPC25’s involvement in this pathway has been well-documented, with studies showing that SPC25 promotes PI3K/AKT activation in tumor cells, contributing to enhanced cell survival and resistance to apoptosis (36, 37, 53) (Figure 2). Study has reported that SPC25 is highly expressed in HCC, and high expression is associated with poor prognosis and metastasis. SPC25 preferentially affects the expression of genes associated with extracellular matrix (ECM) -integrin interactions, including the integrin subunit β4 (ITGB4), an upstream element of the integrin pathway. Up-regulation of ITGB4 partially reversed the decline in cell invasion and migration caused by SPC25 silencing. In addition, deletion of SPC25 and ITGB4 resulted in reduced phosphorylation of focal adhesion kinase (FAK), PI3K, and AKT, which are downstream elements of the integrin pathway. These results demonstrate the important role of SPC25 as a prognostic indicator and metastasis promoter in HCC, suggesting that SPC25 can be used as a biomarker and therapeutic intervention target for HCC treatment (36).

#### 3.2.3 p53 pathway

The tumor suppressor p53 is a critical regulator of cell cycle arrest, DNA repair, and apoptosis. In many cancers, p53 is mutated or functionally inactivated, leading to unchecked cell proliferation (83–85). SPC25 has been found to interact with the p53 pathway, where its overexpression may inhibit p53, contributing to cancer cell survival. For example, in HCC, SPC25 knockdown can significantly reduce the proliferation and metastasis of HCC cells and increase the protein levels of p53 pathway components (70).

## 4 SPC25 as a biomarker and therapeutic target: potential and perspectives

SPC25, as an essential component of the NDC80 kinetochore complex, plays a pivotal role in mitosis, maintaining chromosomal stability, and promoting tumorigenesis. Its dysregulation in various cancers has positioned SPC25 as a promising candidate for both biomarker development and targeted therapy. In this section, we explore the potential of SPC25 as a biomarker for early diagnosis and prognosis, as well as its value as a therapeutic target in cancer treatment.

### 4.1 SPC25 as a biomarker for diagnosis and prognosis

The overexpression of SPC25 in numerous cancers, including breast, lung, gastric, and colorectal cancers, has highlighted its potential as a diagnostic and prognostic biomarker. Elevated SPC25 levels are frequently associated with advanced tumor stages, higher rates of metastasis, and poor OS in cancer patients. Studies have demonstrated that SPC25 is upregulated in tumor tissues compared to adjacent normal tissues, and its expression often correlates with aggressive tumor features, such as larger tumor size and lymph node involvement (17, 86).

For instance, in breast cancer, SPC25 expression has been shown to correlate with high histological grade and poor prognosis (54, 60). In lung cancer, SPC25 levels are positively correlated with tumor size and lymph node metastasis, indicating its potential to serve as a marker for tumor progression (15, 37, 64). In CRC, high SPC25 expression has been linked to poor survival outcomes, reinforcing its prognostic value (13). Given these associations, SPC25 could serve as a reliable biomarker for early detection of aggressive cancers, providing clinicians with valuable information to guide treatment strategies.

In addition, the use of SPC25 as a biomarker for liquid biopsy is also one of the potential areas of research. Liquid biopsies, which involve the analysis of circulating tumor DNA, exosomes, or proteins from blood or other bodily fluids, offer a non-invasive alternative to traditional tissue biopsies (87–89). Given its elevated expression in blood samples from cancer patients, SPC25 could potentially be detected in the circulation, offering a promising tool for monitoring disease progression, recurrence, and response to therapy (90, 91). Liquid biopsy assays targeting SPC25 could provide a real-time snapshot of tumor dynamics, enabling more personalized and timely treatment decisions.

### 4.2 Therapeutic targeting of SPC25: opportunities and challenges

The functional significance of SPC25 in regulating mitosis, chromosomal stability, and cell proliferation presents a compelling rationale for its targeting in cancer therapy. Given its central role in maintaining kinetochore-microtubule attachment during cell division, disrupting SPC25 function could lead to mitotic errors, cell cycle arrest, and apoptosis in rapidly proliferating cancer cells. Several therapeutic strategies are being explored to target SPC25 and its associated pathways, including small molecule inhibitors, RNA interference (RNAi) technologies, and immunotherapies.

#### 4.2.1 Small molecule inhibitors

Small molecules designed to inhibit SPC25 could disrupt its interactions within the NDC80 complex or prevent its binding to microtubules, leading to mitotic defects and cell death. While direct targeting of SPC25 with small molecules remains an area of ongoing research, inhibitors of other mitotic regulators, such as Aurora kinases and Polo-like kinases, have demonstrated some progress (92, 93). These agents exert their effects by disrupting mitotic spindle assembly and promoting chromosomal misalignment, thereby inducing tumor cell death. Given the role of SPC25 in stabilizing the kinetochore-microtubule complex, small molecules targeting SPC25 could potentially mimic these effects, leading to mitotic catastrophe in cancer cells.

#### 4.2.2 RNAi technologies

RNAi technologies, including siRNAs and short hairpin RNAs, offer another promising strategy to target SPC25. By silencing SPC25 expression, RNAi could impair its function, leading to cell cycle arrest and apoptosis (13, 38, 45). Several preclinical studies have demonstrated the effectiveness of RNAi-mediated knockdown of SPC25 in inhibiting cancer cell proliferation and reducing tumor growth (42, 94). The development of RNA-based therapeutics targeting SPC25 could offer a highly specific and effective treatment approach with minimal off-target effects, though challenges in delivery and stability remain.

#### 4.2.3 Antibody-based therapies

Another potential therapeutic approach involves the use of monoclonal antibodies that specifically target SPC25. These antibodies could inhibit SPC25’s interaction with other mitotic machinery components or block its binding to microtubules, ultimately leading to impaired mitosis and cancer cell death. However, developing highly specific and potent antibodies against SPC25 may be challenging, given its involvement in essential cellular processes. Nevertheless, antibody-based therapies targeting other mitotic regulators, such as kinesins or microtubule-associated proteins, have shown promise in preclinical studies, providing a basis for developing similar strategies against SPC25 (95–97).

### 4.3 Clinical perspective: feasibility and challenges

Despite the promising therapeutic potential of targeting SPC25, several challenges remain before its clinical application can become a reality. One major hurdle is the identification of small molecules or biologics that can selectively target SPC25 without affecting other components of the cell cycle machinery, which are essential for normal cell function. Since SPC25 is involved in fundamental processes like mitosis, the risk of off-target effects and toxicity to normal, non-cancerous cells must be carefully evaluated.

Additionally, the development of effective delivery systems for SPC25-targeting agents is crucial. RNA-based therapies, such as siRNA, face challenges related to their stability, delivery to tumor cells, and off-target effects. Nanoparticle-based delivery systems have shown promise in overcoming these challenges by enabling the targeted delivery of RNAi molecules to tumor cells (98, 99). Similarly, small molecules or antibody-based therapies targeting SPC25 will require careful optimization to enhance their specificity, bioavailability, and therapeutic efficacy.

Finally, the heterogeneity of cancer cells and the potential for tumor cells to develop resistance to SPC25-targeting therapies are important considerations. Combining SPC25-targeted therapies with other therapeutic modalities, such as chemotherapy, immunotherapy, or checkpoint inhibitors, may enhance their efficacy and prevent resistance.

## 5 Challenges and future research directions

The growing body of evidence highlighting the role of SPC25 in cancer progression has brought this protein into the spotlight as both a potential biomarker and therapeutic target. However, despite promising data, several challenges remain that limit the full clinical translation of SPC25-targeted strategies. In this section, we discuss the limitations of current research and outline future research directions that could help address these challenges and further elucidate the role of SPC25 in cancer. While considerable progress has been made in understanding the role of SPC25 in various cancers, several limitations in current studies hinder a comprehensive understanding of its function and therapeutic potential.

One of the primary limitations of existing research is the relatively narrow range of cancer types in which SPC25 expression and function have been investigated. Most studies focus on commonly studied cancers such as liver, breast, lung, and colorectal cancers, with limited research on other malignancies like pancreatic cancer, and glioblastoma. The lack of comprehensive data across different cancer types restricts the generalizability of SPC25 as a universal biomarker or therapeutic target. Future studies should focus on expanding the range of cancers examined to provide a more holistic view of SPC25’s role in tumorigenesis and its potential as a target in diverse cancer types.

Although there is growing evidence linking SPC25 to key biological processes such as mitosis and chromosomal stability, the underlying molecular mechanisms remain insufficiently characterized. Many of the studies to date have focused on the correlation between SPC25 expression and tumor progression, but the specific signaling pathways or molecular interactions through which SPC25 exerts its oncogenic effects are not fully understood. For instance, while SPC25 is known to interact with the NDC80 complex and affect microtubule attachment, its precise involvement in modulating key cell cycle regulators and tumor suppressor pathways such as p53, Wnt/*β*-catenin, and PI3K/AKT requires further investigation. More in-depth studies are needed to uncover the detailed molecular networks that SPC25 is part of, which could offer more precise insights into how SPC25 contributes to cancer progression.

Despite the promising potential of SPC25 as a therapeutic target, the development of specific inhibitors against this protein is still in its infancy. Currently, no small molecule inhibitors or monoclonal antibodies specifically targeting SPC25 are available, and much of the preclinical evidence supporting its therapeutic targeting is based on knockdown or overexpression models. The lack of specific and selective inhibitors hampers the development of therapeutic strategies that can be clinically tested. Furthermore, while preclinical studies have demonstrated the potential of SPC25-targeted therapies, there is a significant gap in clinical trials and validation. Without robust clinical evidence, the translation of SPC25-targeted therapies from the laboratory to the clinic remains speculative.

To address these challenges, future research on SPC25 should focus on several key areas to further elucidate its role in cancer and improve its clinical applicability.

Future studies should broaden the scope of cancers examined for SPC25 expression and its clinical significance. In particular, cancers that are less well-studied, such as ovarian, and glioblastoma, should be investigated to determine whether SPC25 holds similar prognostic and therapeutic value. Clinical studies assessing the relationship between SPC25 expression levels and patient outcomes are essential for establishing its role as a reliable biomarker. Longitudinal studies with larger, diverse patient cohorts will be crucial for validating the clinical utility of SPC25 as a biomarker for early detection, prognosis, and therapeutic monitoring.

A critical area for future research is the detailed characterization of the molecular mechanisms through which SPC25 contributes to tumorigenesis. This includes identifying the upstream regulators and downstream effectors of SPC25 and understanding how it interacts with key signaling pathways involved in cancer progression, such as PI3K/AKT, MAPK, and p53. Furthermore, exploring how SPC25 modulates the TME, including interactions with stromal cells, immune cells, and EMC components, will provide valuable insights into its broader role in tumor progression and metastasis. By dissecting the molecular networks in which SPC25 participates, researchers can identify new therapeutic targets and biomarkers that can be used to enhance the efficacy of SPC25-targeted therapies.

Developing specific inhibitors of SPC25 is a critical next step in advancing its potential as a therapeutic target. High-throughput screening of small molecules or the development of monoclonal antibodies targeting SPC25 could lead to the identification of potent and selective inhibitors. These inhibitors could be tested in preclinical models to assess their ability to disrupt SPC25 function and inhibit tumor growth. Additionally, exploring novel drug delivery systems, such as nanoparticles or liposomes, may improve the bioavailability and specificity of SPC25-targeted therapies. Once validated in preclinical models, clinical trials assessing the safety and efficacy of SPC25-targeted therapies in cancer patients will be crucial for translating these findings into therapeutic applications.

The tumor immune microenvironment plays a crucial role in cancer progression and response to treatment, and recent studies suggest that SPC25 may influence immune cell function and immune evasion mechanisms (19, 54). Future research should focus on examining the interactions between SPC25 and various immune cell types within the tumor immune microenvironment, such as tumor-associated macrophages, T cells, and dendritic cells. Understanding how SPC25 modulates immune responses, including its potential to regulate immune checkpoint molecules like programmed cell death ligand 1 or cytotoxic T lymphocyte-associated protein 4, could provide new opportunities for combining SPC25-targeted therapies with immunotherapies, such as immune checkpoint inhibitors. At present, SPC25 has been shown to predict the efficacy of tumor immunotherapy, but the research on SPC25 inhibitors combined with immunotherapy is limited (54). Furthermore, investigating how SPC25 may contribute to chemotherapy and immunotherapy resistance could uncover new strategies to overcome treatment resistance and improve patient outcomes.

Cancer cells often develop resistance to chemotherapy, targeted therapies, and immunotherapies (100, 101), and SPC25 may play a role in this process. Research into the role of SPC25 in drug resistance mechanisms, including its involvement in DNA damage repair, cell cycle regulation, and apoptosis evasion, could provide new insights into how tumors become resistant to treatment. Understanding how SPC25 contributes to the development of resistance to specific therapies will be crucial for designing combination treatment strategies that target SPC25 alongside other resistance pathways.

While SPC25 is one component of the NDC80 complex, its structural features make it particularly attractive as a therapeutic target. Unlike other components such as SPC24 and HEC1, SPC25 has a unique C-terminal domain that directly interacts with microtubules, contributing to the stability of kinetochore-microtubule attachments during mitosis. This direct microtubule-binding ability distinguishes SPC25 from other NDC80 subunits, making it a key player in regulating the dynamics of chromosome segregation (33, 47, 102). Additionally, SPC25’s role in stabilizing the kinetochore-microtubule interface offers potential advantages in targeting mitotic progression, particularly in cancers where microtubule dynamics are disrupted by chemotherapy agents (4, 9). Targeting SPC25 could therefore offer a more specific approach to modulating mitotic functions compared to other NDC80 components, making it a promising candidate for therapeutic intervention in cancer treatment.

Although SPC25 has therapeutic potential in tumors as a key component of the NDC80 complex, its underlying function in normal mitosis may induce off-target toxicity. To improve targeting specificity, potential strategies can be based on the unique biology of cancer cells. The first is synthetic mortality: tumor cells are often accompanied by genomic instability (such as BRCA mutations or deletion of p53), making them more sensitive to microtubule-kinetosomal junction interference. Inhibition of SPC25 may form a synthetic lethal effect with a specific genetic background to selectively kill tumors (such as the synthetic lethal effect of PARP inhibitors and BRCA mutations (103)). Post-translational modification targeting is also one of the directions, but no studies have shown that SPC25-specific post-translational modifications (phosphorylation, ubiquitination) exist in cancer cells and can be used as targets. Local drug delivery or nanocarrier delivery is also a reliable option to limit the accumulation of inhibitors in tumor tissues through tumor microenvironment responsive drug delivery systems (such as PH-sensitive liposomes), reducing the risk of systemic exposure (104). But it’s worth noting. Current studies have not fully clarified the feasibility of the above strategy, and the future needs to be combined with conditional gene knockout models and tumor-specific promoter-driven inhibitor release techniques to verify its *in vivo* selectivity.

While significant progress has been made in understanding the role of SPC25 in cancer, substantial challenges remain in translating these findings into clinical applications. The expansion of cancer type studies, in-depth mechanistic research, and the development of specific inhibitors are all critical for advancing our understanding of SPC25 as a biomarker and therapeutic target. Future research should also focus on the relationship between SPC25 and the immune microenvironment, as well as its involvement in drug resistance mechanisms. By addressing these challenges and expanding the research on SPC25, we can unlock its full potential as a tool for cancer diagnosis, prognosis, and treatment.
